# Transcription factors and potential therapeutic targets for pulmonary hypertension

**DOI:** 10.3389/fcell.2023.1132060

**Published:** 2023-03-17

**Authors:** Liu Yang, Naifu Wan, Fanpeng Gong, Xianfeng Wang, Lei Feng, Guizhu Liu

**Affiliations:** ^1^ Wuxi School of Medicine, Jiangnan University, Wuxi, China; ^2^ Department of Vascular & Cardiology, Ruijin Hospital, School of Medicine, Shanghai Jiao Tong University, Shanghai, China

**Keywords:** pulmonary hypertension, pulmonary vascular remodeling, transcription factors, endothelial cells, pulmonary arterial smooth muscle cells

## Abstract

Pulmonary hypertension (PH) is a refractory and fatal disease characterized by excessive pulmonary arterial cell remodeling. Uncontrolled proliferation and hypertrophy of pulmonary arterial smooth muscle cells (PASMCs), dysfunction of pulmonary arterial endothelial cells (PAECs), and abnormal perivascular infiltration of immune cells result in pulmonary arterial remodeling, followed by increased pulmonary vascular resistance and pulmonary pressure. Although various drugs targeting nitric oxide, endothelin-1 and prostacyclin pathways have been used in clinical settings, the mortality of pulmonary hypertension remains high. Multiple molecular abnormalities have been implicated in pulmonary hypertension, changes in numerous transcription factors have been identified as key regulators in pulmonary hypertension, and a role for pulmonary vascular remodeling has been highlighted. This review consolidates evidence linking transcription factors and their molecular mechanisms, from pulmonary vascular intima PAECs, vascular media PASMCs, and pulmonary arterial adventitia fibroblasts to pulmonary inflammatory cells. These findings will improve the understanding of particularly interactions between transcription factor-mediated cellular signaling pathways and identify novel therapies for pulmonary hypertension.

## 1 Introduction

Pulmonary hypertension (PH) is a chronic life-threatening disorder characterized by progressive pulmonary arterial remodeling (Luks and Hackett, 2022) and inflammatory cell infiltration (Wang R. R. et al., 2022). According to the sixth World Symposium on PH, PH is clinically defined by elevated mean pulmonary arterial pressure (mPAP) >20 mmHg and normal left atrial pressure concomitant with pulmonary vascular resistance ≥3 Wood Units (Simonneau et al., 2019). Structural and functional alterations of pulmonary arterial smooth muscle cells (PASMCs), pulmonary arterial endothelial cells (PAECs), pulmonary arterial fibroblasts, and inflammatory cells contribute to pathological pulmonary vascular remodeling (Southgate et al., 2020). Pulmonary vascular remodeling in PH involves complex mechanisms, including altered crosstalk between the vascular cells, hypoxia, sustained inflammation, resistance to apoptosis, increased proliferation and migration, and excessive activation of signaling pathways (Hassoun, 2021). Uncontrolled proliferation and hypertrophy of disordered pulmonary arterial cells represent hallmark features of pulmonary arterial remodeling. However, the underlying regulatory processes are not well defined.

Three classes of pharmacological therapies are currently available for PH treatment: prostaglandins, endothelin receptor antagonists, and phosphodiesterase type 5 (PDE5) inhibitors. Although these treatments improve pulmonary circulation and reduce hospitalization (Humbert et al., 2022), long-term follow-up shows that they do not reverse pulmonary arterial remodeling or decrease overall PH mortality (Thenappan et al., 2018b). Therefore, new targets and therapies for pulmonary vascular remodeling and PH are urgently required.

Transcription factors are sequence-specific elements that recognize and bind to DNA promoter regions, usually in complex with other proteins, guiding related genome expression. Numerous transcription factors and transcriptional coactivators have been identified as key regulators of PH and pulmonary vascular remodeling. However, a clear landscape of the role of transcription factors in PH progression is needed. Here, we review the involvement of transcription factors in various PH-related cellular mechanisms including in PASMCs, PAECs, pulmonary arterial fibroblasts and inflammatory cells (Table 1), and identify therapeutic targets for PH.

**TABLE 1:** Summary of transcription factors in pulmonary hypertension.

Transcription factors	Targets	Functional cell types	Molecular events and phenotypic features
STAT3	CCNA2	PASMC	STAT3 promotes PASMC proliferation by binding to CCNA2 Zhang et al. (2020) and regulating BMPR2 Brock et al. (2009), NFATc2 Sutendra et al. (2011); Dromparis et al. (2013b), PDGF Qian et al., 2018, c-myc Bai et al. (2006) expressing
	BMPR2		
	NFATc2		
	PDGF c-myc		
	FoxO1		
NFATc2	Kv1.5	PASMC	NFATc2 induces PASMC proliferation and resistance to apoptosis Paulin et al. (2012)
	Bcl2		
Smad2/3	CDKs	PASMC	TGF-β/Smad and BMP/Smad signaling could participated in PASMC proliferation Hata and Chen (2016); Huang and Huang (2005); Gao et al. (2017); Yang et al. (2010)
Smad1/5/8	ID1		
β-catenin	TCF/LEF	PASMC	Wnt/β-catenin signaling contribute to proliferation phenotype of PASMCs Bernkopf et al. (2019)
	CyclinD1		
	c-Myc		
HIF-1α	Notch3	PASMC	HIF-1α functioned as a pathogenic determinant of PASMCs Li et al. (2009)
	Twist1	PAEC	HIF-1α activates Twist1 transcription and regulating EndoMT in hypoxia-induced ECs Zhang et al. (2018a)
	IL-33		IL-33/ST2 enhances PAEC proliferation, adhesion, and angiogenesis Sun et al. (2017); Liu et al. (2018)
			HIF-1α binds to *Twist1* promoter regulating EndoMT in hypoxia-induced ECs Zhang et al. (2018a)
HIF-2α	SPARC	PASMC	HIF-2α induces SPARC expression to increase PASMC proliferation Veith et al. (2022)
		PAEC	EC HIF-2α knockout significantly reduces the progression of hypoxia-induced PH Hu et al. (2019); Cowburn et al. (2016)
			HIF-2α potently promotes EndoMT by contributing to vascular remodeling and NF-kB activation Sun et al. (2020)
FoxO1	p27	PASMC	FoxO1 activation reverses PASMC proliferation and PH development Savai et al. (2014)
	Bcl6		
	CyclinB1/D1		
PPARγ	TGF-β1	PASMC	PPARγ inhibits TGF-β1-induced mitochondrial activation and PASMC proliferation Calvier et al. (2017)
FoxM1	SETD3	PASMC	FoxM1 contributes to pulmonary vascular remodeling by stimulating PASMC dedifferentiation and proliferation Dai et al. (2018a); Dai et al. (2018b); Bourgeois et al. (2018)
	VEGF		FoxM1 promots pyroptosis and proliferation of PASMCs Wu et al. (2022b)
	Survivin		
	KIF23		
	NLRP3 caspase3		
	HMGB1		
KLF2	—	PASMC	Missense mutation in *KLF2* in a family with heritable PH Eichstaedt et al. (2017)
		B cells	A novel missense mutation in KLF2 has been identified in patients with heritable PH Eichstaedt et al. (2017)
KLF5	CyclinB1	PASMC	KLF5 promotes PASMC proliferation Courboulin et al. (2011); Li et al. (2016b)
	HIF-1α		
p53	p21	PASMC	p53 suppress pulmonary vascular remodeling and proliferation under hypoxic conditions. Mizuno et al. (2011); Mouraret et al. (2013)
	HIF-1α	PAEC	p53 was correlated with endothelial dysfunction in PH Alastalo et al. (2011)
	BMPR2		
Runx2	HIF-1α	PASMC	Runx2 promotes PASMC proliferation and apoptosis resistance. Ruffenach et al. (2016)
E2F	CCNA2	PASMC	Pivotal regulators of cell cycle progression Weiss et al. (2019)
	CDK1		
EGR1	LC3B	PASMC	EGR-1 promotes autophagy and alleviated PH progression under hypoxic conditions Lee et al. (2011)
YAP	—	PASMC	YAP aggravats hypoxia-induced autophagy and proliferation of PASMCs Liu et al. (2022)
	MicroRNA-130/301		MicroRNA-130/301 promoting collagen deposition and LOX-dependent remodeling Bertero et al. (2015)
GLI1	ASC	PASMC	GLI1 upregulats ASC by binding to its promoter and triggered pyroptosis in PASMCs He et al. (2020)
ATF6	Nogo-B	PASMC	Decreased ATF6 significantly inducing PASMC apoptosis and reversing pulmonary vascular remodeling Dromparis et al. (2013a)
NFATc3	SM *a*-actin	PASMC	Inhibition of NFATc3 prevents hypoxia-induced increase in SM α-actin expression, arterial wall thickening Gonzalez Bosc et al. (2005); de Frutos et al. (2007); Bierer et al. (2011)
STAT5	—	PASMC	Reduced levels of STAT5 were correlated with the hypertrophy of PASMC in patients with PH Yang et al. (2014)
PPARγ	BMPR2	PAEC	PPARγ regulates genes involved in angiogenesis, survival, and DNA repair, thus maintaining PAEC homeostasis Diebold et al. (2015)
ZNF740	GDF11	PAEC	ZNF740 binds to the GDF11 promoter and promoting PAEC proliferation, angiogenesis, and adhesion molecule expression Yu et al. (2018)
	Smad2/3		
	Smad1/5/8		
TBX4	FGF10	PAEC	TBX4 is a genetic cause of PH, particularly in patients with childhood-onset of the disease Yoshida et al. (2022)
Sox17	HGF c-Met	PAEC	Sox17 deficiency and hypoxia induce excessive EC proliferation, inflammatory activation, and long-lasting PH with high penetrance Park et al. (2022)
Sp1	SHMT2	PAEC	Sp1 promoted PAEC proliferation and inhibited apoptosis by maintaining redox homeostasis Wang et al. (2022b)
FLI1	—	PAEC	Depletion of FLI1 increased EC monolayer permeability, inflammatory cell infiltration, and cytokine expression in the lungs Looney et al. (2017)
Twist1	Tie2	PASMC	Twist1 regulates VSMC proligeration and vascular remodeling Mammoto et al. (2016); Lee et al. (2014)
	PDGF	PAEC	Phosphorylation of Ser42 in Twist1 stimulates EndoMT of ECs through TGF-β/Smad signaling Mammoto et al. (2018)
	TGFBR2		
GATA-6	BMPR2	PAEC	GATA-6 play a critical role in ECs dysfunction in PH, and facilitated PASMC proliferation. Ghatnekar et al. (2013); Fan et al. (2020)
SNAI2	—	PAEC	Upregulated SNAI2 promoted EndoMT of PAECs Hopper et al. (2016)
ZEB1	BMPR1A	PAEC	ZEB1 could prevent excess EndoMT by lowering EC responses to TGF-β stimulation Lee et al. (2022)
SRF/MyoD	α-SMA	PAEC	Cooperation of SRF and MyoD could regulate α-SMA transcription and PAECs transformation Zhang et al. (2018b)
CtBP1	HMOX1	adventitial fibroblasts	Inhibiting CtBP1 decreased glycolysis and inflammatory gene expression in fibroblasts attenuated fibroblast proliferation Li et al. (2016a)
EGR1	—	adventitial fibroblasts	EGR-1 inhibition attenuated the proliferation of adventitial fibroblasts under hypoxic conditions Gerasimovskaya et al. (2002); Banks et al. (2005)
Prx1	TN-C	ECM	Prx1 transactivates the ECM protein tenascin-C (TN-C) gene promoter, promoting SMC proliferation Jones et al. (2001)
CREB	—	ECM	Loss of the transcription factor CREB promoted ECM deposition Klemm et al. (2011)
AP-1	—	ECM	AP-1 controls the cell proliferation, deposition, and turnover of ECM in PH Wagner (2010)
Fra-2/Jun-B	Meprin β	ECM	Fra-2/Jun-B upregulates Meprin β and promotes extracellular matrix components such as collagen in PH Biasin et al. (2014)
Nrf2	MRC1	Macrophage	Nrf2 mediated macrophage phenotype contributing to the development of plexiform lesions in PH Shao et al. (2022)
Hes1	—	PAEC	Decreased Hes1 resulting in increased EC permeability and CD45-positive immune cell infiltration Wang et al. (2022d)
Runx1	p50-NF-κB	macrophage	Runx1 promotes endothelial-to-hematopoietic transformation and pulmonary macrophage recruitment which triggers the production of proinflammatory cytokines Jeong et al. (2022); Luo et al. (2016)
NFATc2	—	T cells	NFATc2 contributing to the inflammatory response of vascular remodeling Bonnet et al. (2007)
NFATc3	COL5A1	T cells	NFATc3 contributed to col V-reactive Th17-mediated immune response in PH Sheak et al. (2020)
AHR	IL-17	ECs bone marrow-derived cells	AHR induced the activation of inflammation and accumulation of CD4^+^IL-21^+^ T cells in the vascular lesions of PH rats Masaki et al. (2021)

## 2 Transcription factors in PASMCs that are associated with PH

PASMCs, located in the medial layer, are the predominant cell type in the arterial wall and play a critical role in maintaining pulmonary vascular homeostasis. The principal function of PASMCs is the regulation of pulmonary circulation and pulmonary arterial pressure. Fully differentiated PASMCs have an extremely low rate of proliferation, and highly express specific contractile proteins and cytoskeleton proteins. The contractile and cytoskeleton proteins, such as smooth muscle (SM) a-actin, SM22α, calponin, SM myosin heavy chain (SM MHC), and myosin light chain (MLC), make up to nearly 40% of the total PASMC protein and are required for PASMC contractile function (Owens, 1995). Of these proteins, SM a-actin, the most widely used marker (Owens et al., 2004), is the first protein expressed during SMC development (Galis et al., 2002). In healthy adults, venous blood flows through the right ventricle and tricuspid valve into the pulmonary artery. The robust contraction of PASMCs maintains pulmonary circulatory pressure at only a quarter of its systemic counterparts. However, as other vascular smooth muscle cells (VSMCs), PASMCs are not terminally differentiated. Following changes such as hypoxia in the pulmonary vascular microenvironment of patients with PH, PASMCs undergo a transition from a contractile phenotype to a pathological state characterized by excessive proliferation, hypertrophy, apoptosis, regulated cell death, cytokine production, and expression of growth factors (Sobue et al., 1999).

### 2.1 Transcription factors involved in the proliferation of PASMCs

**FIGURE 1:**
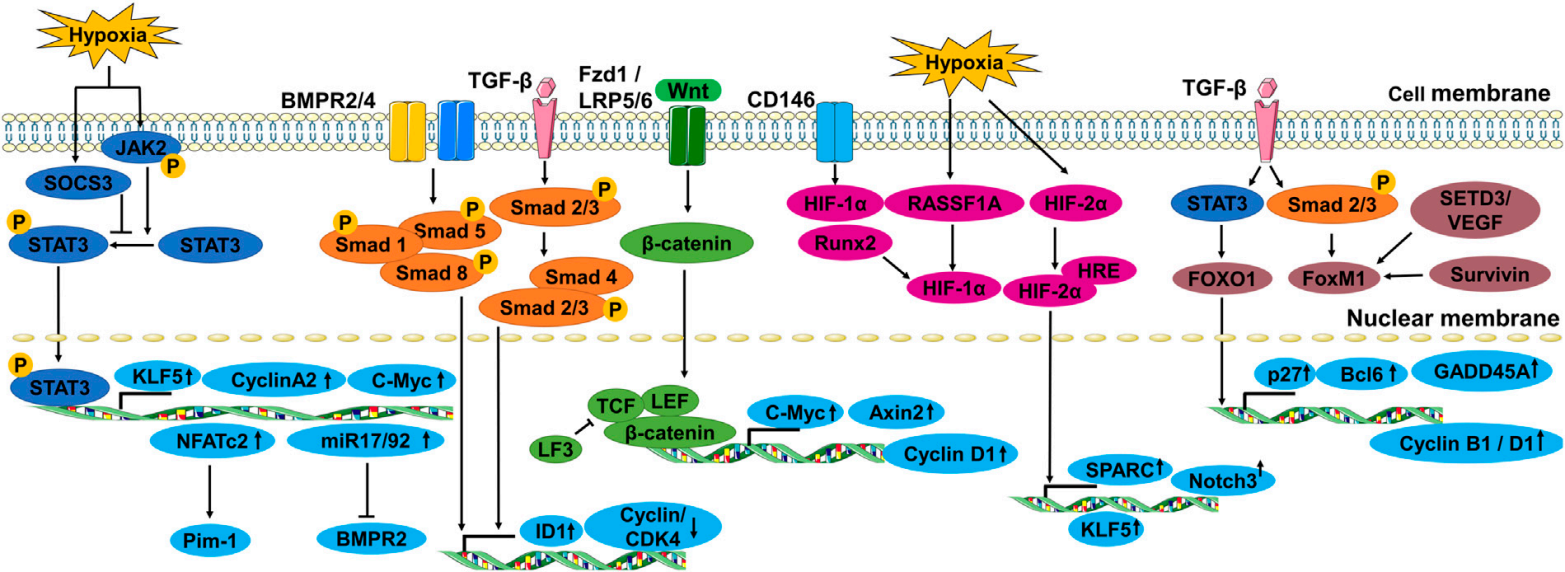
The transcription factors and signal pathways associated with PASMC proliferation in PH.

Pulmonary vascular remodeling is primarily caused by extensive PASMC proliferation, which leads to vascular medial thickening and luminal occlusion. PASMC contraction makes them particularly vulnerable to hypoxia and inflammation. Under these circumstances, PASMCs are comparatively prone to proliferation phenotype alteration, leading to reduced lumen diameters and increased peripheral resistance (Tajsic and Morrell, 2011). Chronic hypoxia-induced medium thickening can be accounted for proliferation of a specific subpopulation of SMCs within the media (Stenmark et al., 2006). Here, we summarize the multiple transcription factors and pathways associated with PASMC proliferation in PH (Figure 1).

#### 2.1.1 STAT3 transcription factor

Janus kinase (JAK)/signal transducer and activator of transcription (STAT) is a canonical membrane-to-nucleus pathway which can be activated by various cytokines, interferons (IFNs), and growth factors (O'Shea et al., 2015). Recent studies have shown that the JAK2/STAT3 pathway plays a significant role in PASMC proliferation and PH development. Hypoxia stimulates the phosphorylation of JAK2 and the activation of STAT3, resulting in the translocation of STAT3 to the nucleus. STAT3 binds to the promoter region of *CCNA2* (encodes cyclin A2) and transcriptionally activates cyclin A2, promoting PASMC proliferation and PH. SMC-specific JAK2 deficiency or JAK inhibitors effectively reversed the excessive, hypoxia-induced PASMC proliferation and pulmonary vascular remodeling (Zhang et al., 2020). Moreover, ruxolitinib, the U.S. Food and Drug Administration-approved JAK1 and JAK2 inhibitor, effectively inhibited the proliferation and migration of PASMCs in healthy individuals and patients with idiopathic pulmonary arterial hypertension (IPAH). Accordingly, ruxolitinib attenuated pulmonary vascular remodeling and the elevation of pulmonary arterial pressure both in monocrotaline (MCT)-induced PH rats and hypoxia-induced PH mice in a dose-dependent manner by blocking the JAK2/STAT3 pathway, suggesting a novel therapeutic option for PH patients (Yerabolu et al., 2021). Bone morphogenetic protein receptor 2 (BMPR2), a membrane protein receptor belonging to the transforming growth factor (TGF) family, mediates the binding of bone morphogenetic proteins (BMPs) that have been identified as inhibitors of PASMC proliferation. Loss-of-function mutations in BMPR2 account for approximately 75% of all PH-related abnormalities in patients with a known family history of PH (Soubrier et al., 2013). Downregulated BMPR2 expression has also been described in non-heritable forms of PH (Atkinson et al., 2002). STAT3 downregulates BMPR2 expression by activating microRNA (miR)-17/92 transcription, contributing to PH development. Inhibiting STAT3 activation by anti-cytokine therapies against IL-6 might provide a feasible alternative therapy for restoring the BMPR2 function and preventing PH development (Brock et al., 2009). A phase II clinical trial on the satralizumab, an anti-IL-6 receptor antibody in the treatment of PH is ongoing (NCT05679570), which may provide novel therapeutic option for PH patients. Nuclear factor of activated T cells (NFAT), a transcription factor that mediates adaptive T-cell functions, also regulates innate immunity and vascular cells in the pulmonary microenvironment (Fric et al., 2012). NFATc2 induces PASMC proliferation and resistance to apoptosis in the remodeling pulmonary arteries by inhibiting the expression of Kv1.5 and upregulating the expression of anti-apoptotic protein B-cell leukemia/lymphoma-2 (Bcl2) (Paulin et al., 2012). Interestingly, the expression of NFATc2 and the subsequent proliferation of PASMCs can be regulated by STAT3, which binds to the NFATc2 promoter region and positively regulates the NFATc2 activator, Pim-1, an oncoprotein specifically high expressed in PH (Paulin et al., 2011).

The STAT3 pathway in PASMCs is regulated by many biomolecules under hypoxic conditions, leading to proliferation-related gene activation in the nucleus. For example, platelet-derived growth factor (PDGF) is a key causative factor of PH progression that induces the hyperproliferation of PASMCs. miR-1181 inversely regulates STAT3 protein levels by interacting directly with the 3′-UTR of STAT3 in human PASMCs, while PDGF-BB downregulates miR-1181 expression. The miR-1181/STAT3 axis functions downstream of the PDGF pathway to regulate PASMC proliferation and migration (Qian et al., 2018). In addition, suppressor of cytokine signaling 3 (*SOCS3*) has been identified as a hypoxia-inducible gene that suppresses tyrosine phosphorylation of STAT3 and transcription of the *c-myc* gene, inhibiting PASMC proliferation stimulated by hypoxia in a classical negative feedback loop (Bai et al., 2006).

#### 2.1.2 Smad signaling

Another predominant transcription factor family involved in the proliferation of PASMCs is Smad. Multiple growth factors and cytokines have been reported to activate PASMC proliferation during vascular remodeling of PH (Akhurst and Hata, 2012). Transforming growth factor β (TGF-β) is a multifunctional cytokine that is increased in PH animal models. Different members of the Smad family have different roles in signaling. TGF-β receptors (TGFBRs) activate Smad2 and Smad3, whereas BMP4 receptors activate Smad1, Smad5, and Smad8. Once activated, Smad2/3 or Smad 1/5/8 form a complex with Smad4 and is translocated to the nucleus where it directly regulates the transcription of multiple target genes. These pathways have numerous functions in cell proliferation (Hata and Chen, 2016). TGF-β/Smad signaling can block cell transition from the G1 to S phase, a process known as growth arrest, by downregulating the activity of cyclin-dependent kinases (CDKs) that mediate the cell cycle (Huang and Huang, 2005). Under hypoxic conditions, excess TGF-β levels initiate cell proliferation *via* TGFBR-mediated activation of Smad2/3, which forms a complex with Smad4 and translocates to the nucleus to regulate multiple target genes (Gao et al., 2017). Osthole reportedly inhibits the TGF-β/Smad signaling pathway, thus decreases cyclin/CDK4 expression and induces cell cycle arrest at the G0/G1 phase. As a result, excessive PASMC proliferation and MCT-induced rat PH are prevented (Yue et al., 2020). Inhibitors of the DNA binding (ID) family of proteins, comprising IDs 1 to 4, are major transcriptional targets of BMP/Smad signaling and are involved in BMP/Smad-induced suppression of PASMC proliferation. Targeting the BMP/Smad pathway using the prostacyclin analogues iloprost and treprostinil inhibited the proliferation of PASMCs and prevented PH progression by enhancing Smad1/5 and ID1 signaling (Yang et al., 2010). Intriguingly, berberine, a natural compound that has been used in Chinese medicines for many years, increased the expression of BMPR2 and its downstream p-Smad1/5, and decreased the expression of TGF-β and its downstream p-Smad2/3, thus protecting against hypoxia-induced PASMC proliferation and vascular remodeling (Chen et al., 2019). Notably, prolonged exposure to TGF-β can result in loss of Smad3 (Yanagisawa et al., 1998). Smad3 depletion increases proliferation and migration of PASMCs, although this can be attenuated by inhibiting myocardin-related transcription factor (MRTF) (Zabini et al., 2018). Moreover, the clinical trials showed that inhibiting Smad2/3 signaling with sotatercept, a novel fusion protein binding activins, effectively reduced pulmonary vascular resistance in PH patients with longer-term safety and durability (Humbert et al., 2021; Humbert et al., 2023).

#### 2.1.3 Wnt/β-catenin signaling pathway

The Wnt/β-catenin signaling pathway plays a prominent role in maintaining pulmonary vascular homeostasis by accelerating postinjury healing (Nusse and Clevers, 2017), but is also closely correlated with the pathogenesis of PH. Wnt proteins are a large family of secreted glycoproteins that signal by binding to Frizzled receptor 1 (Fzd1) and low-density lipoprotein receptor-related protein 5/6 (LRP5/6). Abnormal Wnt activation reduces the degradation of β-catenin by destroying the complex in the cytoplasm, resulting in β-catenin accumulation in the cytoplasm. Then, β-catenin translocates to nuclei and binds T-cell factor (TCF)/lymphoid enhancer factor (LEF) to promote the expression of downstream genes, including Cyclin D1 (*CCND1*), *c-Myc*, *Axin2*, and *LEF1* (Bernkopf et al., 2019). Activation of Wnt/β-catenin signaling also induces PASMC proliferation and vascular remodeling by inducing the expression of the downstream positive effectors Cyclin D1 and c-Myc. LF3, a small molecule, blocks the interaction between β-catenin and TCF4 and inhibits the downstream gene expression, thus preventing the proliferation and migration of PASMCs in rats with PH (Lei et al., 2021). We also recently revealed that resolvin E1 (RvE1), a pro-resolving lipid mediator, suppresses hypoxia-induced PASMC proliferation and experimental PH by inhibiting proliferative Wnt7a/β-catenin signaling (Liu et al., 2021). Thus, Wnt/β-catenin signaling in PASMCs may act as a therapeutic target for managing pulmonary vascular remodeling and PH.

#### 2.1.4 Hypoxia-inducible transcription factors

Pulmonary hypoxic response is an evolutionarily conserved stress response triggered by reduced availability of oxygen in the alveolar (Luo et al., 2019). Hypoxia plays an essential role in PH and is a well-established independent cause of vascular remodeling and PH (Sylvester et al., 2012). Hypoxia-inducible factors (HIFs) act as key regulators of oxygen homeostasis and hypoxic adaptation in the lungs. During PH development, aberrant HIF-1α activation functions as a pathogenic determinant of PASMC expansion and pulmonary vascular remodeling (Semenza, 2012; Wilkins et al., 2015). A recent study showed that CD146 expression and HIF-1α transcription reinforce each other to physiologically promote PASMC hyperproliferation. Disruption of the CD146/HIF-1α crosstalk in SMCs through genetic ablation of CD146 or anti-CD146 antibodies mitigates pulmonary vascular remodeling and enhances cardiac function in different PH models, providing a proof-of-concept for anti-remodeling PH therapy (Luo et al., 2019). In addition, Ras-association domain family 1 A (RASSF1A) is stabilized by hypoxia and thus bound to HIF-1α, preventing hydroxylation of its prolyl group, promoting nuclear translocation and subsequent transcriptional activity. This, in turn, leads to increased RASSF1A levels, resulting in a feed-forward loop and increasing proliferation and glycolysis during PH. RASSF1A ablation protects against hypoxia-induced pulmonary vascular remodeling and right ventricle hypertrophy, and this effect can be exerted in heterozygous mice (Dabral et al., 2019). Notch proteins are cell membrane receptors that are involved in vascular morphogenesis and function. Notch3 is a downstream target of hypoxia and is upregulated by HIF-1α. Compared with wild-type and Notch3^+/−^ mice, Notch3^−/−^ mice did not develop PH even after exposure to chronic hypoxia for 6 weeks, and showed no muscularization of the small pulmonary arteries and arterioles, no hypertrophic remodeling, and no VSMC proliferation (Li et al., 2009). HIF-2α bound to hypoxia response elements (HRE) in secreted protein acidic and rich in cysteine (SPARC) promoter and induce SPARC expression in hypoxia-induced PASMCs. Inhibiting SPARC enhanced apoptosis, reduced the proliferation of PASMCs, and significantly improved hemodynamic and cardiac function in mice with PH (Veith et al., 2022).

#### 2.1.5 FoxO1 and FoxM1

FoxO transcription factors are a family of transcriptional regulators containing a conserved DNA-binding domain forkhead box (Accili and Arden, 2004). FoxOs can act as transcriptional activators and repressors when located in the nucleus and bound to promoters that contain the FoxO consensus motif. Studies have reported that FoxOs are implicated in various cellular responses and vascular structural maintenance (Oellerich and Potente, 2012; Eijkelenboom and Burgering, 2013). FoxO1 and its downstream apoptosis-related target genes *p27*, B-cell lymphoma six protein (*BCL6*), and DNA damage-inducible protein 45 (*GADD45A*) are downregulated in the pulmonary vasculature of patients with IPAH as well as in rats with PH, while proliferative cyclin B1 and cyclin D1 are upregulated, indicating a hyperproliferative and apoptosis-resistant PASMC phenotype. Constitutive FoxO1 activation by adenovirus, psammaplysene (a small-molecule activator of FoxO1) or paclitaxel (an antineoplastic agent that induces FoxO1 expression and activity) efficiently reverses PASMC proliferation and PH development, indicating a potential therapeutic potential for PH intervention (Savai et al., 2014). A pro-proliferative TGF-β1-STAT3-FoxO1 axis has also been identified in hypoxia-induced PASMCs. Peroxisome proliferator-activated receptor gamma (PPARγ), is a nuclear receptor that functions as a transcription factor to regulate adipogenesis, metabolism, and PH development (Lehrke and Lazar, 2005; Legchenko et al., 2018). PPARγ inhibits TGF-β1-induced mitochondrial activation and PASMC proliferation, whereas the PPARγ agonist pioglitazone reverses PH progression by inhibiting the TGFβ1-STAT3-FoxO1 axis in TGF-β1-overexpressing mice (Calvier et al., 2017).

Forkhead box M1 (FoxM1), a multifunctional transcription factor in the Forkhead family that promotes cell proliferation, angiogenesis, epithelial-mesenchymal transition, and DNA damage repair (Myatt and Lam, 2008; Yang et al., 2018), also plays a crucial role in the pathogenesis of PH (Gu and Liu, 2020). FoxM1 expression is elevated in PASMCs of both PH patients and animal models, stimulating PASMC dedifferentiation and proliferation. Induction of SMC-specific FoxM1 deficiency or pharmacological inhibition of FoxM1 can reverse existing pulmonary vascular remodeling and inhibit PH progression, suggesting that suppressing FoxM1 might be a promising therapeutic strategy for PH (Dai J. et al., 2018; Dai Z. Y. et al., 2018; Bourgeois et al., 2018). Multiple signaling pathways, including HIFs, TGF-β/Smad, SET domain-containing 3 (SETD3)/vascular endothelial growth factor (VEGF), survivin, cell cycle regulatory genes, and DNA damage response (DDR) networks, cross-talk with FoxM1 during PASMC proliferation and PH progression (Gu and Liu, 2020).

#### 2.1.6 Kruppel-like factors (KLFs)

Kruppel-like factors (KLFs) belong to a family of zinc finger transcription factors and play dominant roles in the cardiovascular, immune, respiratory, digestive, and hematopoietic systems (McConnell and Yang, 2010). Accumulating evidence has implicated KLF signaling in PH pathogenesis. For example, a recent study identified a missense mutation in *KLF2* that disrupted gene function in a family with heritable PH (Eichstaedt et al., 2017). KLF5 expression was upregulated in both human lungs and cultured PASMCs isolated from patients with IPAH, and was correlated with disease severity. Inhibiting the STAT3 pathway abrogates KLF5 activation in hypoxia-induced PASMCs. Once activated, KLF5 promotes cyclin B1 upregulation and PASMC proliferation (Courboulin et al., 2011). KLF5 also acts as an upstream regulator of HIF-1α and promotes hypoxia-induced PASMC proliferation and migration, as well as resistance to apoptosis in a HIF-1α-dependent manner, providing a better understanding of PH pathogenesis (Li X. et al., 2016).

#### 2.1.7 Other transcription factors

The growth-suppressive and pro-apoptotic transcription factor p53 plays a key role in PASMC proliferation and PH. It interacts with p21 and HIF-1α to suppress pulmonary vascular remodeling and proliferation under hypoxic conditions (Mizuno et al., 2011). Stabilizing p53 with Nutlin-3 had therapeutic effects against PH by limiting PASMC proliferation (Mouraret et al., 2013). Runt-related transcription factor 2 (Runx2), the key mediator of vascular calcification, is upregulated in lung, distal pulmonary arteries, and primary PASMCs isolated from PH patients, driven by miR-204 downregulation. Runx2 further triggers HIF-1α activation and enhances its nuclear localization, thus promoting PASMC proliferation and apoptosis resistance. Runx2 inhibition improved pulmonary hemodynamic and pulmonary vascular remodeling in rats with Sugen/hypoxia-induced PH (Ruffenach et al., 2016). The HIPPO signaling pathway is a key regulator of the proliferation/apoptosis balance to prevent organ overgrowth. Large tumor suppressor 1 (LATS1) is the central component of HIPPO pathway and is inactivated in PASMCs of IPAH patients, which upregulates proliferative/pro-survival signaling and promotes PASMC proliferation during PH. Pharmacological inhibition of integrin-linked kinase 1 (ILK1) upregulates LATS1, inhibits proliferation, induces apoptosis in PASMCs of individuals with PH, and reverses established pulmonary vascular remodeling and PH progression (Kudryashova et al., 2016). Using a congenic breeding program and comparative genomics to study two rat strains that differ in their response to chronic hypoxia, researchers identified the gene *Slc39a12*, which encodes the zinc transporter ZIP12, as a master regulator of hypoxia-induced pulmonary vascular remodeling. Inhibition of ZIP12 expression disrupted PASMC proliferation and attenuated PH development in rats housed under hypoxic conditions. The fundamental role of zinc transporters in mammalian pulmonary arterial homeostasis suggests a novel therapeutic target for PH management (Zhao et al., 2015).

Studies have also focused on transcription factors that are directly associated with cell cycle progression and cellular proliferation in PASMCs of individuals with PH (Weiss et al., 2019). For example, E2F transcription factors are pivotal regulators of cell cycle progression *via* the target genes *CCNA2* (encodes Cyclin A2) and *CDK1* (encodes cyclin-dependent kinase 1), which drive cells through the S phase. Inhibiting CDK using palbociclib and dinaciclib inhibits PASMC proliferation by arresting the cell cycle and interfering with the E2F signaling pathway, thus reducing pulmonary vascular remodeling in PH (Weiss et al., 2019).

### 2.2 Transcription factors involved in the death of PASMCs

**FIGURE 2:**
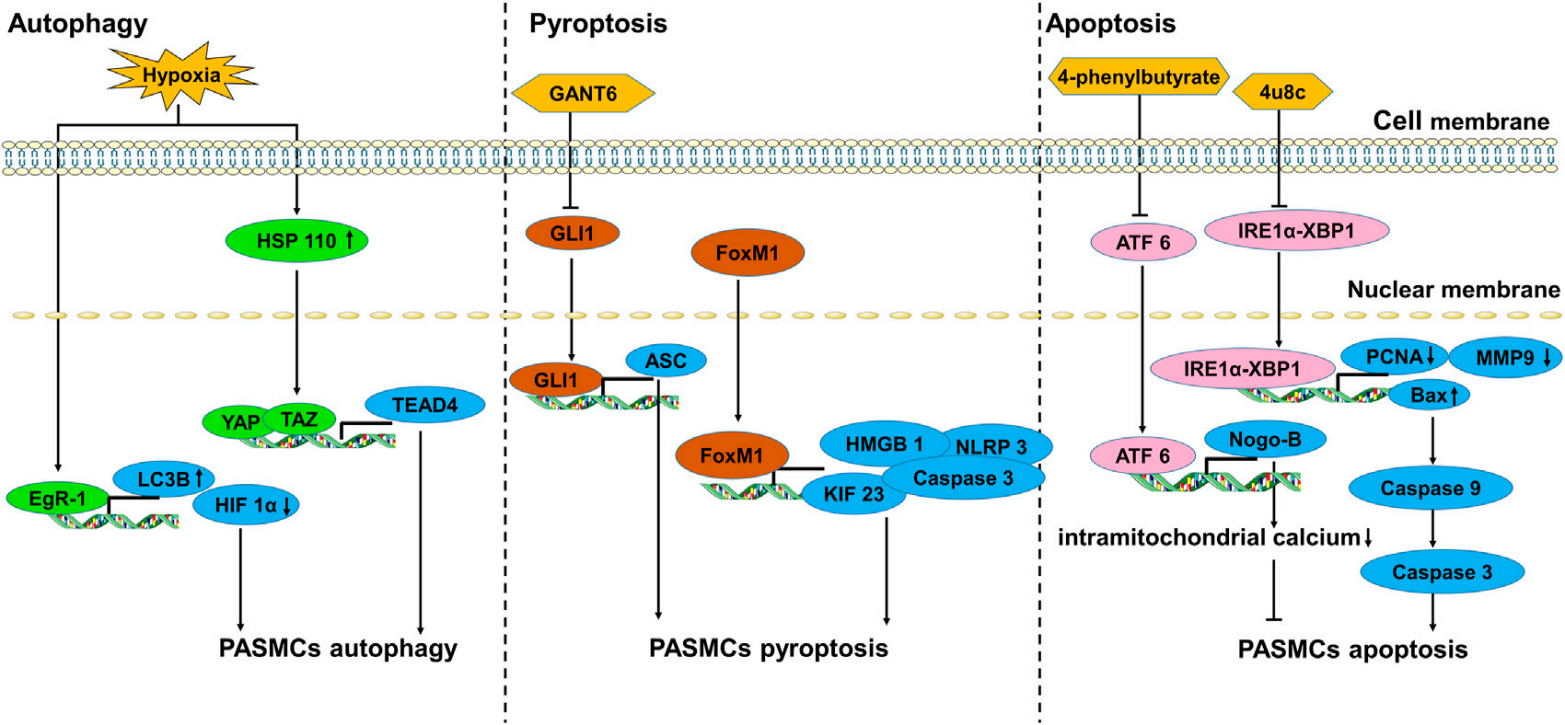
The transcription factors involved in the death of PASMCs.

In hypoxic environments, PASMCs undergo phenotypic transformation to aberrant proliferation and apoptosis resistance. Different forms of programmed cell death (PCD) of PASMCs, including autophagy (Lee et al., 2011; Wu et al., 2019), pyroptosis (Shi et al., 2017) and mitochondrion-dependent apoptosis (Sutendra et al., 2011) have also been reported in PH (Figure 2).

#### 2.2.1 Transcription factors associated with PASMC autophagy

Autophagy, represented by the conversion of microtubule-associated protein-1 light chain 3 B (LC3B) from free form LC3B-I to phosphatidylethanolamine conjugated form LC3B-II and autophagosome formation, was increased in hypoxia-treated PASMCs and lung vasculature of patients with PH. Mechanistically, the early growth response-1 (EGR-1) transcription factor was rapidly induced to bind to the LC3B promoter upon hypoxic stimulation, and the upregulated LC3B suppressed reactive oxygen species (ROS)-dependent stabilization of HIF-1α. LC3B and EGR-1 deficiency reduced autophagy and aggravated PH progression under hypoxic conditions, suggesting the involvement of autophagy in pulmonary vascular remodeling (Lee et al., 2011). However, studies also found that autophagy contributes to BMPR2 degradation and PH development (Gomez-Puerto et al., 2019). Inhibition of autophagy and lysosomal BMPR2 degradation by chloroquine prevents MCT-induced PH progression in rats (Long et al., 2013). Similarly, liraglutide, a glucagon-like peptide-1 (GLP-1) receptor agonist, attenuates PASMC autophagy to ameliorate PH by inhibiting the mitochondrial fusion protein dynamin-related 1 (DRP1) and autophagy-related protein (Atg)-5/Atg-7/Beclin-1/LC3B pathway (Wu et al., 2019). Heat shock protein-110 (HSP110), a chaperone with anti-aggregation properties, plays a role in multiple human diseases. HSP110 expression increased significantly in the pulmonary arteries and PASMCs of mice under hypoxic conditions. Knocking down HSP110 inhibited yes-associated protein (YAP) and transcriptional coactivator with PDZ-binding motif (TAZ) activity and TEAD4 nuclear expression, alleviating hypoxia-induced autophagy and proliferation of PASMCs, right ventricle systolic pressure, vascular wall thickening, and right ventricular hypertrophy in mice (Liu et al., 2022).

#### 2.2.2 Transcription factors involved in the pyroptosis of PASMCs

Pyroptosis is a recently discovered and confirmed inflammatory form of cell death (de Gassart and Martinon, 2015). It plays a role in diverse cardiovascular disease processes (He et al., 2020; Shi et al., 2021; Wu Y. et al., 2022). Inflammatory caspases are pivotal for pyroptosis, and include caspase-1-mediated canonical inflammasome and caspase-4/5/11-mediated non-canonical inflammasome pathways (Kovacs and Miao, 2017). Caspase-1, the key effector of the inflammasome, mediates inflammation by activating the proinflammatory cytokines interleukin (IL)-18 and IL-1β. Impaired inflammation and SMC proliferation were observed in hypoxic caspase1^−/−^ mice compared with WT controls (Udjus et al., 2019). Notably, glioma-associated oncogene family zinc finger 1 (GLI1), a ubiquitously distributed transcriptional activator that participates in multiple diseases, is aberrantly expressed under hypoxic conditions and is mainly distributed in PASMCs in the pulmonary arteries. GLI1 upregulates apoptosis-associated speck-like protein containing a caspase recruitment domain (ASC) that plays a crucial role in the activation of pyroptosis in PASMCs by binding to the ASC promoter region. The GLI1-specific inhibitor, GANT61, reversed pyroptotic cell death and attenuated hypoxia-induced pulmonary vascular remodeling and PH development (He et al., 2020). Furthermore, KIF23, the target gene of FoxM1 (Li et al., 2022), is a nuclear protein that regulates cytokinesis and microtubule binding. A recent study identified KIF23 as the most important gene in IPAH and pyroptosis, and its expression was markedly upregulated in PASMCs of rats with IPAH. KIF23 interacts with and regulates the expression of NLRP3, Caspase3, and HMGB1, thus promoting pyroptosis and proliferation of PASMCs. Knocking down KIF23 reduced pyroptosis in PASMCs and alleviated IPAH in rats (Wu Z. et al., 2022).

#### 2.2.3 Transcription factors associated with mitochondria-dependent apoptosis of PASMCs

Endoplasmic reticulum (ER) stress and mitochondria-dependent apoptosis participate in PH development. Calcium from the ER, the largest cellular Ca^2+^ store, enters the mitochondria to promote the opening of the mitochondrial transition pore, resulting in cytochrome c release to initiate mitochondria-dependent apoptosis (Zamzami and Kroemer, 2001). The metabolic modulator dichloroacetate (DCA) prevents and reverses MCT-induced PH in rats by inducing mitochondria-dependent apoptosis and reversing the downregulation of Kv1.5 (McMurtry et al., 2004). Nogo-B, a regulator of the ER structure, is induced in PASMCs and pulmonary arteries during PH. Nogo-B disrupts the mitochondria-ER unit and decreases intramitochondrial calcium, leading to mitochondrial hyperpolarization and suppression of PASMC apoptosis (Sutendra et al., 2011). ER stress response involves three pathways: protein kinase R [PKR]-like ER kinase (PERK), activating transcription factor 6 (ATF6), and inositol-requiring enzyme 1α (IRE1α)-X-box binding protein 1 (XBP1). The transcription factor ATF6 is activated in hypoxic and PH PASMCs, leading to the induction of Nogo-B expression. Clinical chemical chaperones, 4-phenylbutyrate (PBA) or tauroursodeoxycholic acid, decreased ATF6 activation and induced PASMC apoptosis, significantly reducing and reversing pulmonary vascular remodeling in PH mice and rats (Dromparis et al., 2013a). The IRE1α-XBP1 pathway is also markedly upregulated in hypoxic PASMCs. 4u8c, an inhibitor of the IRE1α-XBP1 pathway, increased hypoxia-induced cell apoptosis by downregulating the expression of PCNA and MMP9, and activated mitochondrial apoptosis by upregulating BAX and activating caspase-9 and caspase-3 (Cao et al., 2019). Taken together, these observations show that targeting the key transcription factors involved in ER stress may be a novel therapeutic strategy for PH.

### 2.3 Transcription factors associated with PASMC hypertrophy

PASMC hypertrophy manifests as increased cell size, appearing to be a consequence of increased intracellular protein and water content (Berk, 2001). NFAT, a Ca^2+^-dependent transcription factor, has recently been linked to the maintenance of the smooth muscle phenotype. NFATc3 and serum response factor (SRF) bind to a region of the first intron of the SM *a*-actin gene, an SMC hypertrophic marker, enhancing its expression in cultured aortic smooth muscle cells (Gonzalez Bosc et al., 2005). NFATc3 transcriptional activity and nuclear translocation were activated in PASMCs and pulmonary arteries of both adult and neonatal mice following exposure to hypoxic conditions. Pharmacological inhibition or genetic ablation of NFATc3 prevents chronic hypoxia-induced increase in SM *a*-actin expression, arterial wall thickening (de Frutos et al., 2007), right ventricular hypertrophy and PH in mice (Bierer et al., 2011). Reduced levels of the transcription factor STAT5 are correlated with the hypertrophy of PASMCs in patients with PH (Yang et al., 2014).

## 3 Transcription factors associated with pulmonary arterial endothelial cells (PAECs) during PH

In addition to PASMCs, endothelial cells (ECs) also play a dominant role in the initiation and progression of PH. Dysfunctional EC signaling is responsible for many of the characteristics of PH, including pulmonary inflammation, oxidative stress, vascular cell apoptosis resistance, proliferation, metabolic reprogramming, and immune cell accumulation (Evans et al., 2021). Genetic ablation of Egln1 (encoding prolyl-4 hydroxylase-2, PHD2) in ECs and bone marrow cells have severe PH and recapitulate many features of clinical PH in mice, including occlusive vascular remodeling and right heart failure, indicating the critical role of EC dysfunction in PH pathogenesis (Dai et al., 2016). Similarly, mice administered SU5416, the VEGF receptor antagonist, or rats treated with MCT develop PH that resembles human disease. In these models, endothelial apoptosis occurs in the first 3 days of injury; however, endothelial cells become hyperproliferative and apoptosis resistant by day seven (Sakao et al., 2005; Sauler et al., 2019). Several transcription factors are associated with ECs in the pathogenesis of PH.

### 3.1 Transcription factors involved in endothelial dysfunction

There is abundant evidence supporting the crucial involvement of HIF transcription factors in the functional alteration of PAECs other than PASMCs. Disordered proliferation and angiogenesis of PAECs are important stages in the development of PH. VEGF is a key “end effector” molecule in the vascular remodeling of PH that promotes angiogenesis by regulating EC proliferation, migration, and differentiation (Thompson and Lawrie, 2017). HIF-1α, a “molecular switch,” regulates VEGF expression. HIF-1α interacts with the promoter region of IL-33, resulting in the induction of IL-33 expression (Sun et al., 2017). Expression of IL-33 and its membrane receptor ST2 was significantly upregulated in lung tissues of mice and patients with PH. IL-33/ST2 enhances PAEC proliferation, adhesion, and angiogenesis through the HIF-1α/VEGFA axis, while ST2 knockout attenuates pulmonary vascular remodeling and PH development (Liu et al., 2018). A recent study found that HIF-2α activity in ECs, which is required for the initiation of hypoxia-induced PH, is activated very early following exposure to hypoxic conditions. Inflammatory factors and proliferation genes were upregulated in ECs in an HIF-2α (but not HIF-1α)-dependent manner in response to acute hypoxia. A small-molecule inhibitor of HIF-2α, PT2567, or EC HIF-2α knockout significantly reduced the progression of hypoxia-induced PH in adult rats (Hu et al., 2019). Likewise, EC deletion of arginase-1, another downstream target of HIF-2α, attenuated many of the pathophysiological symptoms associated with hypoxic PH. Chronic hypoxia enhanced HIF-2α stability, increasing arginase expression and dysregulating normal vascular NO homeostasis (Cowburn et al., 2016). In addition, HIF-2α interacted with p53 to accelerate EC apoptosis under hypoxia, resulting in the dysfunction of pulmonary arterial endothelium (Wang et al., 2019). Of note, Nutlin3a-mediated p53 induction could inhibit PASMC proliferation and PH development (Mouraret et al., 2013), but it would also enhance PAEC apoptosis and exert a potentially pathogenic effect on PH. Thus, a combination of the p53 agonist Nutlin3a and the HIF-2α antagonist PT2385 confers greater therapeutic efficacy against PH, than monotherapy with either agent alone (Zheng et al., 2022).

Numerous studies have linked PAEC dysfunction in PH to mutations or dysfunctions in BMPR2 (Lane et al., 2000; Teichert-Kuliszewska et al., 2006). Specifically, two transcription factors, PPARγ and tumor suppressor p53, mediate gene regulation downstream of BMPR2 in PAECs and are correlated with endothelial dysfunction in PH (Alastalo et al., 2011; Diebold et al., 2015). In addition, BMPR2 signaling is particularly prominent in maintaining DNA integrity in PAECs under hypoxic conditions (Li et al., 2014). Interestingly, PPARγ and p53 form a DNA damage-dependent transcription factor complex that regulates genes involved in angiogenesis, survival, and DNA repair, thus maintaining PAEC homeostasis. Conditional BMPR2 deletion in ECs promoted DNA damage and PAEC apoptosis and impaired the reversal of hypoxia-associated PH, similar to mice with PPARγ deletion in ECs (Alastalo et al., 2011; Diebold et al., 2015). However, stabilization of p53 with Nutlin-3 could rescueimpaired PPARγ-p53 complex formation and reversed PH in EC-BMPR2 deficient mice (Hennigs et al., 2021). Hypoxia also decreased PPARγ and miR-98 expression, increasing the levels of endothelin-1 (ET-1), which stimulates PAEC proliferation and contributes to pulmonary vascular remodeling and PH. Activation of PPARγ using rosiglitazone attenuated PAEC proliferation and hypoxia-induced PH by restoring miR-98 levels (Kang et al., 2016). Growth differentiation factor 11 (GDF11), also known as BMP11, is a member of the BMP/TGF-β superfamily. During PH, the transcription factor zinc finger protein 740 (ZNF740) activates GDF11 expression, thus activating Smad2/3 and Smad1/5/8 signaling in PAECs and promoting PAEC proliferation and angiogenesis. EC-specific GDF11 knockout or ZNF740 inhibition attenuates pulmonary vascular remodeling and PH (Yu et al., 2018). Mediator complex subunit 1 (MED1) is a key transcriptional coactivator and KLF4 is a master transcription factor in the endothelium. MED1 acts synergistically with KLF4 to transactivate BMPR2, ERG, and TGFBR2 *via* chromatin remodeling and enhancer-promoter interactions in PAECs. MED1 levels were decreased in the lung tissue and PAECs of patients and mice with PH, while MED1 overexpression mitigated the PH phenotype in rodents (Wang C. et al., 2022).

Notch signaling has been implicated in vascular development, homeostasis, and injury responses. The expression and transcriptional activity of Notch1 in PAECs increases under hypoxic conditions. Inhibiting Notch1 increased p21 expression and the Bax/Bcl-2 ratio, thus significantly reducing PAECsproliferation and apoptosis resistance during PH (Dabral et al., 2016). Notch2 promotes endothelial apoptosis and its expression was attenuated in PAECs exposed to vasoactive stimuli, including hypoxia, TGF-β, and ET-1. Decreased levels of Notch2 increased E2F1 binding to the Notch1 promoter and upregulated Notch1 expression, promoting PAEC proliferation and resistance to apoptosis and resulting in endothelial dysfunction and subsequent PH-associated vascular remodeling (Sahoo et al., 2021).

TBX4*,* a member of the TBX family of proteins containing a T-box DNA-binding domain, is a transcription factor crucial for lung development (Papaioannou, 2014). Genetic researches have reported that *TBX4,* located on chromosome 17q23.2, is a genetic cause of PH, particularly in patients with childhood-onset of the disease (Nimmakayalu et al., 2011; Kerstjens-Frederikse et al., 2013). Fibroblast growth factor 10 (FGF10) is essential for fetal lung development. *TBX4* directly regulates FGF10 through a consensus T-box binding site, whereas the TBX4 variants (*p*.L257 T fs*129 and *p*. R126C) do not activate FGF10 and fail to induce maturation of the pulmonary vascular endothelium, a potential cause of PH (Yoshida et al., 2022).

Large-scale genomic studies have suggested that SRY-related HMG-box17 (Sox17), an endothelial-specific transcription factor that regulates EC and vascular homeostasis, is a putative causal gene for PH (Graf et al., 2018; Hiraide et al., 2018; Zhu et al., 2018; Rhodes et al., 2019). Indeed, Sox17 deficiency and hypoxia induce excessive EC proliferation, inflammatory activation, and long-lasting PH with high penetrance. Mechanistically, Sox17 deficiency activates hepatocyte growth factor (HGF) and its receptor c-Met, which promotes cell growth. Sox17 dysfunction and reactive upregulation of HGF/c-Met signaling may act as novel druggable targets for PH treatment (Park et al., 2022).

Dickkopf 1 (DKK1), a secretory glycoprotein, plays a pathologic role in the development of PH. Increased DKK1 levels in PAECs during PH activate the transcription of Serine Hydroxymethyltransferase 2 (SHMT2) *via* the transcription factor specificity protein 1 (Sp1), promoting PAEC proliferation and inhibiting apoptosis by maintaining redox homeostasis. Endothelium-specific knockdown of DKK1 or DKK1 neutralizing antibodies significantly ameliorate PH progression (Wang Q. Q. et al., 2022).

The E-twenty-six (ETS) family of transcription factors is a prominent regulator of the gene network maintaining endothelial homeostasis. The expression of Friend leukemia integration one transcription factor (FLI1), a ETS family member, and its closest homolog, ETS-related gene (ERG), were significantly downregulated in ECs in lungs of patients and mice with PH. Depletion of ERG or FLI1 increased EC monolayer permeability, inflammatory cell infiltration, and cytokine expression in the lungs. Thus, the loss of ERG or FLI1 in ECs may contribute to the pathogenesis of PH through induction of inflammation (Looney et al., 2017).

### 3.2 Transcription factors associated with endothelial-to-mesenchymal transition (EndoMT)

Endothelial-to-mesenchymal transition (EndoMT) is a biological process through which ECs acquire a mesenchymal or myofibroblastic phenotype. During this process, ECs lose their cell-cell junctions and gain migratory and invasive abilities that enable them to reach surrounding tissues. During migration, the cells express reduced levels of specific endothelial markers such as CD31 and VE-cadherin, and progressively express mesenchymal or myofibroblastic markers, including *a*-SMA and vimentin (Frid et al., 2002). Cells with a mixed mesenchymal/endothelial phenotype are present in the intimal and plexiform lesions of PH lungs, and EndoMT is a critical mechanism of pulmonary vascular remodeling responsible for PH both in human and experimental PH animals (Ranchoux et al., 2015). Transcriptional factors such as Twist-1, HIFs, SNAIs, and high-mobility group AT-hook 1 (HMGA1) in PAECs have been revealed as the master factors initiating EndoMT during PH pathology.

The transcription factor Twist1 controls the expression of various angiogenic factors and receptors such as Tie2 (Mammoto et al., 2016) and PDGF (Lee et al., 2014), which control VSMC proliferation and vascular remodeling. Twist1 also cross-talks with HIF-1α (Yang and Wu, 2008), Wnt (West et al., 2014), and TGF-β (Cufi et al., 2010), which mediate the pathogenesis of PH. Twist1 expression is enhanced in the lungs of PH patients. Twist1 binds to the TGFBR2 promoter region to promote its transcriptional activity. Phosphorylation of Ser42 in Twist1 stimulates EndoMT of ECs through TGF-β/Smad signaling, while knocking down Twist1 attenuates hypoxia-induced pulmonary arterial remodeling and PH progression in mice (Mammoto et al., 2018). HIF-1α is also essential for hypoxia-induced EndoMT. Interestingly, HIF-1α was found to bind to the *Twist1* promoter, thus activating *Twist1* transcription and regulating EndoMT in hypoxia-induced ECs (Zhang B. et al., 2018). GATA-6, a member of the GATA family of zinc-finger transcription factors, plays a critical role in EC dysfunction in PH (Ghatnekar et al., 2013). BMPR2 has been recognized as a novel downstream target of GATA-6, which directly binds to the BMPR2 promoter. Twist1 promotes the ubiquitination-proteasomal degradation of GATA-6, thus repressing BMPR2 transcription and facilitating PASMC proliferation and migration (Fan et al., 2020). Taken together, these findings show that inhibiting Twist1 may constitute a new therapeutic strategy for PH.

The SNAI zinc-finger transcription factors, including SNAI1, SNAI2, and SNAI3 (also known as SNAIL, SLUG, and SMUC, respectively), has been reported to play a central role in the progression of EndoMT (Kokudo et al., 2008). Loss of BMPR2 in PAECs leads to increased HMGA1 expression, which upregulates SNAI2 and promotes EndoMT of PAECs (Hopper et al., 2016). HIF-2α transcriptionally upregulates SNAI1 and SNAI2, subsequently triggering EndoMT in ECs of patients with PH (Liu et al., 2011; Tang et al., 2018). Furthermore, HIF-2α potently increases EC nicotinamide phosphoribosyl transferase (NAMPT) promoter activity and promotes EndoMT. Inhibiting NAMPT significantly reduced MCT-induced vascular remodeling, NF-κB activation, and PH development, providing a novel and attractive therapeutic target for PH vascular remodeling (Sun et al., 2020).

In addition to BMPR2, BMPR1 is also important for maintaining endothelial identity and preventing excess EndoMT. The transcription factor ZEB1 is the primary target of BMPR1A. Following BMPR1A activation, ID2 physically interacts with and sequesters ZEB1 to inhibit the transcription of TGFBR2, in turn lowering EC responses to TGF-β stimulation and preventing excess EndoMT. BMPR1A-ID2/ZEB1-TGFBR2 signaling may serve as a promising therapeutic target for PH and other EndoMT-related vascular disorders (Lee et al., 2022).

The expression of peroxisome proliferator-activated receptor gamma coactivator 1-alpha (PGC-1α) was recently shown to be suppressed in PAECs of patients with PH as well as in hypoxia-induced PH mice. PGC-1α overexpression rescued the expression of endothelial nitric oxide synthase (eNOS) and blocked EndoMT of PAECs during PH (Cai and Chen, 2022). In addition, Galectin-3, a member of the *ß*-galactoside-binding gene family, facilitated the cooperation between transcription factor SRF and myogenic differentiation 1 (MyoD), regulating *a*-SMA transcription and PAEC transformation (Zhang L. et al., 2018).

## 4 Transcription factors that are activated in fibroblasts and extracellular matrix (ECM) during PH

The extracellular matrix (ECM) comprises all extracellular constituents that are crucial for tissue structure, function, and intercellular communication. The ECM is necessary for maintaining tissue homeostasis under both physiological and pathophysiological conditions, and too little or too much ECM production can result in severe dysfunction. Alterations in the vascular ECM are increasingly being recognized as key drivers of PH. Fibroblasts are the predominant producers of ECM (DeLeon-Pennell et al., 2020). Activated fibroblasts are prone to secreting extra ECM, which interferes with pulmonary vascular homeostasis under hypoxic environments in lung vasculature of individuals with PH. Robust proliferation of fibroblasts and accumulation of macrophages were observed in the pulmonary artery adventitia in the early stage of PH (Das et al., 2008; Li et al., 2011; El Kasmi et al., 2014). Metabolic reprogramming has been suggested to contribute to the aberrant phenotype of fibroblasts in PH. C-terminal binding protein 1 (CtBP1), the NADH-sensitive transcriptional corepressor, is a potential sensor of the cellular metabolic state that modulates the cell phenotype (Chinnadurai, 2009). The anti-inflammatory gene, *HMOX1*, is a direct target of CtBP1. Inhibiting CtBP1 or NADH decreased glycolysis and inflammatory gene expression in fibroblasts attenuated fibroblast proliferation and suppressed macrophage accumulation in patients with PH (Li M. et al., 2016). Cultured pulmonary artery fibroblasts showed increased expression and DNA-binding activity of the EGR-1 transcription factor in response to hypoxia. Directly inhibiting EGR-1 attenuated the proliferation of adventitial fibroblasts under hypoxic conditions (Gerasimovskaya et al., 2002; Banks et al., 2005).

ECM remodeling and pulmonary vascular stiffness occur early in the PH process, even before the increase of intimal and medial thickness and pulmonary arterial pressure, suggesting a causal role rather than a consequence of ECM in pulmonary vascular remodeling (Thenappan et al., 2018a). Pharmacological inhibition of vascular ECM constituents such as serine elastase, lysyl oxidase (LOX), and periostin, can reverse pulmonary vascular remodeling and ameliorate PH development (Cowan et al., 2000; Nave et al., 2014; Nie et al., 2020). Indeed, ECM remodeling and stiffness may trigger the proliferation of adventitial fibroblasts, ECs, and VSMCs through mechanoactivation of various transcription factors and signaling pathways. The transcriptional coactivators YAP and TAZ are important for mechanotransduction, which converts extracellular mechanical cues into intracellular signals (Dupont et al., 2011). It has recently been proposed that YAP and TAZ activation by ECM stiffening is a molecular driver of PH (Bertero et al., 2015; Bertero et al., 2016). ECM stiffening induces the microRNA-130/301 family by activating YAP/TAZ. MicroRNA-130/301 controls the PPARγ-ApoE-LDL receptor-related protein 8 (LRP8) axis, promoting collagen deposition and LOX-dependent remodeling. Therapeutic targeting of the downstream ApoE or LOX suppresses YAP/TAZ-miR-130/301 circuit and ameliorates ECM remodeling and PH (Bertero et al., 2015). Furthermore, ECM stiffening promoted vascular cell growth and migration through YAP/TAZ-dependent glutaminolysis and anaplerosis. The finding links mechanical stimuli to dysregulated vascular metabolism and identifies potential metabolic drug targets for developing PH therapeutics (Bertero et al., 2016). Expression of the homeobox transcription factor Prx1 is upregulated during PH and transactivates the ECM protein tenascin-C (TN-C) gene promoter, promoting SMC proliferation and PH progress (Jones et al., 2001). Mammalian Ste20-like kinases (MSTs) 1/2 are members of the HIPPO pathway that act as growth suppressors of proliferative diseases in adults. However, in contrast to canonical antiproliferative/proapoptotic roles, MST1/2 acts as a pro-proliferative/prosurvival molecule in human pulmonary arterial adventitial fibroblasts by downregulating FoxO3 and promoting established pulmonary vascular remodeling in PH mice (Kudryashova et al., 2022).

In addition, oxidative stress contributes to pulmonary arterial remodeling by stimulating the loss of the transcription factor CREB and promoting SMC growth and ECM deposition. The oxidant scavenger Tempol effectively attenuates chronic hypoxia-induced CREB loss, excessive elastin/matrix production, and pulmonary arterial remodeling (Klemm et al., 2011). The activator protein-1 (AP-1) complex, a dimeric transcription factor composed of Fos (c-Fos, Fra-1, Fra-2, FosB) and Jun (c-Jun, Jun-B, Jun-D) proteins has been implicated in the control of cell proliferation, deposition, and turnover of ECM (Wagner, 2010). Fra-2 transgenic mice are novel models of PH associated with systemic sclerosis (Maurer et al., 2012). Mechanistically, the Fra-2/Jun-B complex binds to the meprin β promoter, leading to upregulated expression. Meprin β promotes vascular remodeling in PH by acting on extracellular matrix components such as collagen (Biasin et al., 2014).

## 5 Transcription factors activated in immune cells during PH

In recent years, greater attention has been paid to the frequently observed perivascular inflammation in PH patients. Inflammatory and autoimmune processes are increasingly being recognized as major pathogenic factors of pulmonary arterial remodeling. Pulmonary vascular lesions in patients and animals with PH are characterized by varying degrees of perivascular inflammatory infiltrates, including macrophages, T- and B-lymphocytes, dendritic cells (DCs), and mast cells (Savai et al., 2012). In addition to increased perivascular immune cell accumulation and intravascular infiltration, high levels of various cytokines and chemokines, including IL-1β, IL-6, IL-8, monocyte chemoattractant protein 1 (MCP-1), chemokine ligand 5 (CCL5), and tumor necrosis factor (TNF)-α, have been identified in PH animals and patients. These cytokines and chemokines are correlated with poor clinical outcomes in patients with PH and may serve as potential biomarkers of PH progression (Rabinovitch et al., 2014). Concomitantly, vascular cells, including ECs, PASMCs, and fibroblasts, may also change their phenotypes, resulting in enhanced capacity to stage inflammatory responses and increased secretion of cytokines and chemokines. Transcription factors play prominent roles in immune cells and inflammatory responses during PH.

### 5.1 Transcription factors in macrophages

Macrophages are the most abundant inflammatory cells in lung tissue (Reyfman et al., 2019). Early and persistent accumulation of macrophages has been observed in perivascular lesions in PH patients as well as animals (Vergadi et al., 2011). Altered macrophage polarization can induce experimental PH (Zawia et al., 2021). Interventions targeting macrophages, for example macrophage depletion, have confirmed their causal roles in PH and pulmonary vascular remodeling (Talati et al., 2016; Zaloudikova et al., 2016).

Adventitial fibroblasts derived from hypertensive pulmonary arteries activate macrophages through paracrine IL-6 and STAT3, HIF-1α, and C/EBPβ signaling to drive the expression of genes involved in inflammatory response, tissue remodeling, and PH. Targeting IL-6 signaling in macrophages by completely inhibiting C/EBPβ or HIF-1α or by partially inhibiting STAT3 presents therapeutic value for treating PH (El Kasmi et al., 2014). Right ventricular function is considered a major determinant of cardiac capacity and survival of patients with PH. The NLRP3 inflammasome pathway within the right ventricular macrophages was activated in two robust preclinical PH models and in patients with PH. Blocking the IL-6/STAT3 pathway suppressed macrophage accumulation and NLRP3 inflammasome activation, thus improving cardiac function in PH rats (Al-Qazazi et al., 2022).

Nuclear factor erythroid 2-related factor 2 (Nrf2), a stress-responsive transcription factor, binds to the promoter of the macrophage lineage marker, mannose receptor C-type 1 (MRC1), to trigger its expression. Thus, Nrf2 mediates bone marrow-derived early progenitor endothelial cell (eEPC) transformation to the macrophage phenotype, contributing to the development of plexiform lesions in PH (Shao et al., 2022). Impaired Notch1 cleavage in PAECs decreases the expression of the downstream transcription factor Hes1, resulting in increased EC permeability and CD45-positive immune cell infiltration in PH mouse models (Wang S. M. et al., 2022). Direct binding of the transcription factor Runx1 to p50-NF-κB in macrophages triggers the production of proinflammatory cytokines (Luo et al., 2016). Furthermore, Runx1 promotes endothelial-to-hematopoietic transformation and pulmonary macrophage recruitment and activation during PH progression. Genetic ablation of Runx1 in adult endothelium or in myeloid lineage cells protected mice against developing PH and reversed established PH and improved vascular remodeling in rats (Jeong et al., 2022).

### 5.2 Transcription factors in T cells

T cell-deficient athymic rats developed particularly severe PH (Miyata et al., 2000; Taraseviciene-Stewart et al., 2007), whereas immune reconstitution prevented early accumulation of macrophages and B cells, attenuated pulmonary endothelial apoptosis, and upregulated BMPR2 expression, thus preventing experimental PH (Tamosiuniene et al., 2011). Similarly, regulatory T cell (Treg) treatment significantly decreased pro-inflammatory cytokine expression and enhanced the expression of vasoprotective proteins such as cyclooxygenase 2 (COX-2), programmed death ligand 1 (PDL-1), and heme oxygenase 1 (HO-1), thus protecting against PH development (Chu et al., 2015; Tamosiuniene et al., 2018). The fact that inflammation precedes vascular remodeling suggests a causal role of altered immunity in PH progression.

NFAT, a Ca2^+^/calcineurin-sensitive transcription factor, is a key T cell activator and plays a central role in the pathogenesis of PH. NFATc2 is activated in the pulmonary arteries and in circulating CD3-positive T lymphocytes, contributing to the inflammatory response of vascular remodeling. NFAT inhibition reversed the effects of established PH in rats (Bonnet et al., 2007). NF-κB is activated in the pulmonary arterial lesions of PH patients and rats. Inhibiting NF-κB using pyrrolidine dithiocarbamate (PDTC) mitigated and prevented pulmonary arterial obliteration in rats by increasing perivascular CD4^+^ T cells (particularly Tregs) and reducing perivascular CD8^+^ T lymphocytes and CD45RA^+^ B lymphocytes (Farkas et al., 2014). The protective role of sulfasalazine, an NF-κB inhibitor, is being verified by a phase I clinical trial (NCT04528056).

The T helper 2 (Th2) cell immune response is also implicated in pulmonary vascular remodeling during PH progression. The expression of a chemoattractant receptor homologous molecule expressed on Th2 cells (CRTH2) was upregulated in circulating CD3^+^CD4^+^ T cells in PH patients and animals. CRTH2 promoted Th2 cell activation, inducing PASMC proliferation through STAT6 activation. Targeting the CRTH2-mediated Th2 response provides a potential therapeutic strategy for PH (Chen et al., 2018). Chronic hypoxia-induced PH partly results from T helper 17 (Th17)-mediated perivascular inflammation. NFATc3 is an important transcription factor for chronic hypoxia-induced PH in adult mice. Study finds that NFATc3 directly regulates the expression of the lung self-antigen *COL5A1*, the gene encoding the α1-helix of collagen type V (col V), contributing to col V-reactive Th17-mediated immune response in PH (Sheak et al., 2020). The aryl hydrocarbon receptor (AHR), a nuclear receptor/transcription factor, mediates multiple immune diseases by regulating inflammatory signals, including cytokine and chemokine signals (Stockinger et al., 2014). AHR promotes IL-17-producing Th17 differentiation (Nakahama et al., 2013). The IL-6/Th17/IL-21 axis plays a prominent role in a hypoxia-induced PH in mouse models by promoting M2 macrophage polarization (Hashimoto-Kataoka et al., 2015). Moreover, AHR in both ECs and bone marrow-derived cells induced the activation of inflammation and accumulation of CD4^+^IL-21^+^ T cells in the vascular lesions of PH rats in the advanced stages. The AHR signaling pathway may be a novel therapeutic target for PH, and AHR agonistic activity in the serum is a PH biomarker (Masaki et al., 2021).

### 5.3 Transcription factors in B cells

B cells play a vital role in lung hemostasis. Pulmonary injury, together with enhanced B-cell activation, is sufficient for inducing PH symptoms in mice, and adaptive immune activation contributes to IPAH induction or progression (Heukels et al., 2021). Simultaneously, IPAH patients have a distinct RNA expression profile of peripheral blood B-lymphocyte compared with that of healthy controls, with several genes being clearly upregulated (Ulrich et al., 2008). Elimination of B lymphocytes with anti-CD20 antibody prevented the increase of HIF-1α and VEGF and reduced mean pulmonary arterial pressure in mice with PH (Mizuno et al., 2012). A novel missense mutation in the transcription factor KLF2, which has been described as a recurrent somatic mutation in B-cell lymphoma, has also been identified in patients with heritable PH (Eichstaedt et al., 2017). These findings will improve our understanding of the multifunctional roles of B cells in PH.

## 6 Conclusion and perspectives

**FIGURE 3:**
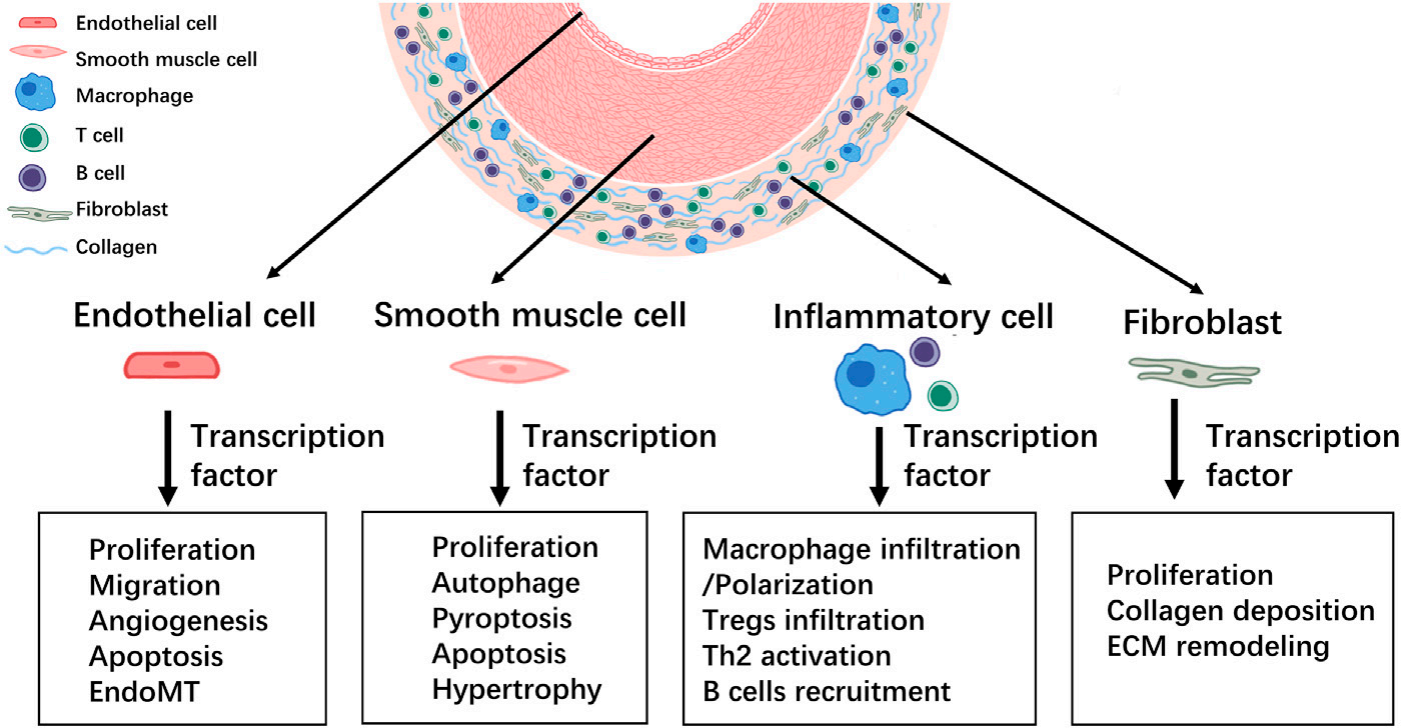
Summary of the involvement of transcription factors in the development of pulmonary vascular remodeling during PH.

Pulmonary vascular remodeling is characterized by medial hypertrophy/hyperplasia, intimal and adventitial fibrosis, plexiform lesions, and perivascular infiltration of immune cells (Humbert et al., 2019). It is well established that complex interactions among cells and signaling pathways are involved in the development of pulmonary vascular remodeling. The original concept that PH is largely caused by vasoconstriction has expanded over the last few decades. However, it is now accepted that therapeutic strategies are supposed to address not only vasodilation, but also pulmonary vascular remodeling, by inhibiting proliferative mechanisms to reduce PH mortality. Transcription factors and associated signaling pathways play predominant roles in pulmonary vascular remodeling and PH pathogenesis (Figure 3). Approaches targeting single cytokines, growth factors, or receptor tyrosine kinases may generate limited efficacy because of the complicated stimuli implicated in pulmonary vascular remodeling. One promising strategy is to target the prominent downstream effector molecules such as transcription factors that integrate various signal pathways. Although multiple transcription factors and their associated therapeutic roles in PH have been suggested, further in-depth and comprehensive research in this field is needed to identify new opportunities for strategic PH intervention and drug development. Moreover, further investigations are required to evaluate the clinical applicability of PH therapies targeting transcription factors.
